# Multivariate spatio-temporal approach to identify vulnerable localities in dengue risk areas using Geographic Information System (GIS)

**DOI:** 10.1038/s41598-021-83204-1

**Published:** 2021-02-18

**Authors:** Gayan P. Withanage, Malika Gunawardana, Sameera D. Viswakula, Krishantha Samaraweera, Nilmini S. Gunawardena, Menaka D. Hapugoda

**Affiliations:** 1 Molecular Medicine Unit, Faculty of Medicine, University of Kelaniya, Ragama, Sri Lanka; 2 Postgraduate Institute of Science, University of Peradeniya, Peradeniya, Sri Lanka; 3 Department of Statistics, Faculty of Science, University of Colombo, Colombo, 07 Sri Lanka; 4 Epidemiology Unit, Office of the Regional Director of Health Services, Gampaha, Sri Lanka

**Keywords:** Infectious diseases, Ecological modelling

## Abstract

Dengue is one of the most important vector-borne infection in Sri Lanka currently leading to vast economic and social burden. Neither a vaccine nor drug is still not being practiced, vector controlling is the best approach to control disease transmission in the country. Therefore, early warning systems are imminent requirement. The aim of the study was to develop Geographic Information System (GIS)-based multivariate analysis model to detect risk hotspots of dengue in the Gampaha District, Sri Lanka to control diseases transmission. A risk model and spatial Poisson point process model were developed using separate layers for patient incidence locations, positive breeding containers, roads, total buildings, public places, land use maps and elevation in four high risk areas in the district. Spatial correlations of each study layer with patient incidences was identified using Kernel density and Euclidean distance functions with minimum allowed distance parameter. Output files of risk model indicate that high risk localities are in close proximity to roads and coincide with vegetation coverage while the Poisson model highlighted the proximity of high intensity localities to public places and possibility of artificial reservoirs of dengue. The latter model further indicate that clustering of dengue cases in a radius of approximately 150 m in high risk areas indicating areas need intensive attention in future vector surveillances.

## Introduction

Dengue is an arthropod-borne viral infection found the throughout tropical and subtropical regions of the world. Based on publish literatures, World Health Organization (WHO) estimates that about 390 million dengue virus infections occurs every year of which 96 million manifest clinically^1,2^. The infection has spread over 128 countries globally while 3.9 billion people are at risk and approximately 70% of the incidences are reported from Asia^3^.

Dengue is the most important vector-borne emerging and re-emerging infectious disease in Sri Lanka at present. The first serologically confirmed dengue incidence was reported in Sri Lanka in 1962 followed by an increase in the number of dengue incidences over the time^4^. The first dengue epidemic was reported in the country in 1989 and since then, dengue was considered to be endemic in the country and periodic dengue fever (DF) and dengue hemorrhagic fever (DHF) epidemics progressively occurred with increased magnitude^5^. In Sri Lanka, more than 30,000 dengue cases have been reported to the Epidemiology Unit of Ministry of Health since 2010 while more than half of the cases reported in the Western Province. The second highest prevalence of dengue is observed in the Gampaha District in the Western province over 10 years^6^. The largest dengue epidemic in Sri Lanka was reported in 2017 with a total of 186,101 dengue incidences. Moreover, this is considered as the worst mosquito-borne virus infection in the South Asian countries^7^. During the peak of the epidemic in July, 2017, the highest number of incidences were reported from the Gampaha District^6^. In the absence of an effective drug or vaccine specific to the dengue virus (DENV), source reduction is the best method to control transmission of dengue. In addition to conventional vector surveys, chemical control methods such as selective indoor residual insecticide spraying and insecticide treated nets (ITNs) were currently using to control the disease transmission^8^. Recently, an autocidal gravid ovitrap have been developed using Novaluron as the active ingredient to control the dengue transmission in the district^9^. Thermal fogging is using in Sri Lanka as the last option in vector control methods with portable or vehicle mounted fog generators. Even though, ideally fogging should be implemented every 2–3 days for 10 days, fogging is repeated within 7–10 days after initial spraying in Sri Lanka^10^. Organophosphates and synthetic pyrethroid insecticides were using national-wide in fogging since Sri Lanka has history a history of extensive use of DDT which has already led higher resistance in dengue vector mosquito species^11^. Deltamethrin, Lambda-cyhalothrin, Malathion, Permathrin, and *d*,*d*,*trans*-cyphenothrin are currently using in aerosol applications and fogging Sri Lanka^12^.

In recent years, health authorities directed their attention toward development of early warning systems to reduce disease transmission rates in high risk areas. Predictive risk maps, developed using Geographical Information System (GIS)-based approaches and spatial statistical analysis, may provide an alternative approach to identify risk localities assessing and quantifying the distribution of risk factors to predict impending dengue epidemics^13^. Further, GIS-based risk maps can be used to understand the mosquito distribution, seasonal climatic variations and environmental factors affecting transmission of dengue^14,15^.

Currently, GIS-based risk predictive models are not available yet for the Gampaha District to assess to identify risk localities and these models are needs of the moment to control dengue transmission. Therefore, the objective of the present study is to analyse spatial and seasonal distribution of dengue incidence and ecological factors to develop GIS-based risk model for the identification of risk localities in high-risk areas in order to control dengue transmission. The outcome of the study will probably improve the effectiveness of dengue surveillance programmes, ultimately controlling impending dengue epidemics in the district.

## Results

Risk maps were prepared to identify both the risk areas in the Gampaha District and distribution of dengue incidences and risk factors in the selected dengue high risk study areas.

### Identification of risk localities in areas with high risk using the developed GIS-based model

**Figure 1:**
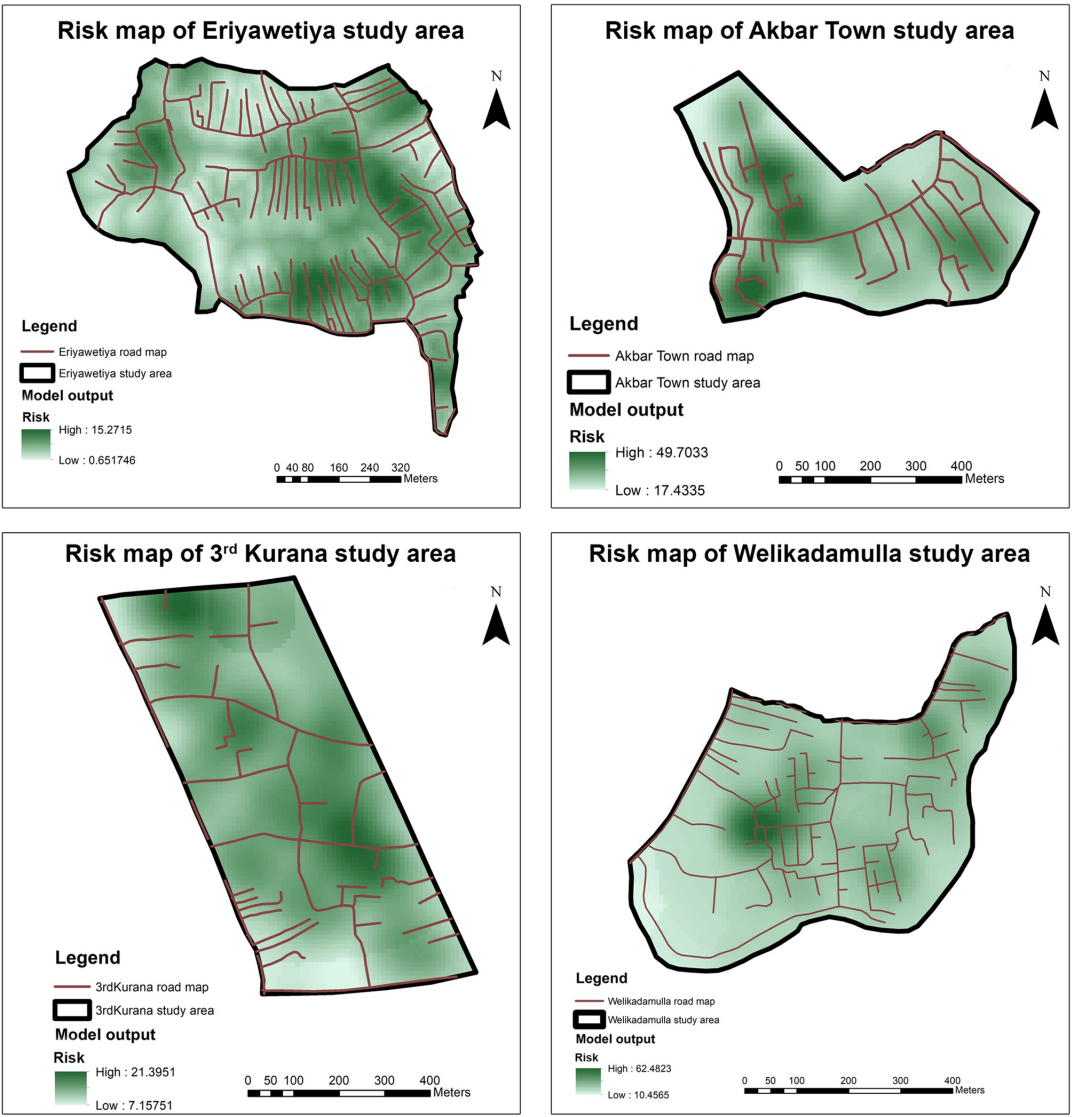
Generated risk map for the study areas. High risk localities were illustrated in the dark green colour while the low risk localities were illustrated in white. Road maps were overlapped in the respective area. Satellite imagery comparisons were illustrated in Supplementary Figures S1–S4. Risk maps were composed using Esri ArcGIS 10.2.1.

The WGS 84 / UTM zone 44 N georeferenced layers were used for the development of GIS-based model and the developed model was utilized to identify the risk localities in the dengue high risk study areas in the Gampaha District. The final outcomes of the model for each study area were depicted in Fig. 1. The comparisons of model outputs with the satellite images of respective high risk areas were illustrated in the Supplementary Figs. S1–S4.

### Identifications of spatial correlations between different layers with patient locations in each dengue high risk study area in the Gampaha District

A random point layer was generated by introducing 100 random points for each study area separately. The generated random point layer was used to extract spatial distances for each study raster and spatial correlations were analysed using the Pearson's product correlation coefficient^16^. The results of the correlations were summarized in the Table 1.

**Table 1:** Spatial correlations between generated layers with dengue incident location layer in each dengue high risk study area in the multivariate Poisson point process model.

Study layer	Correlations in each study area			
	Eriyawetiya	Akbar Town	3^rd^ Kurana	Welikadamulla
Positive breeding locations	0.04	0.14	0.16	0.36*
Contour	− 0.03	0.15	0.18	− 0.16
Roads	0.03	0.34*	0.27*	0.45*
Total buildings	− 0.02	− 0.43*	− 0.10	− 0.44*
Public places	0.04	− 0.38*	− 0.07	− 0.35*
Land use-urban areas	0.11	− 0.01	N/A	0.21*
Land use-home gardens	− 0.05	− 0.18	0.16	− 0.45*
Land use-marshy lands	0.08	− 0.15	0.16	− 0.18

*Significantly correlated layers with dengue incidences during the study period. A 5% significance level was used to identify significant correlations. N/A-Urban areas are not included in the land use maps of 3^rd^ Kurana study area.

**Figure 2:**
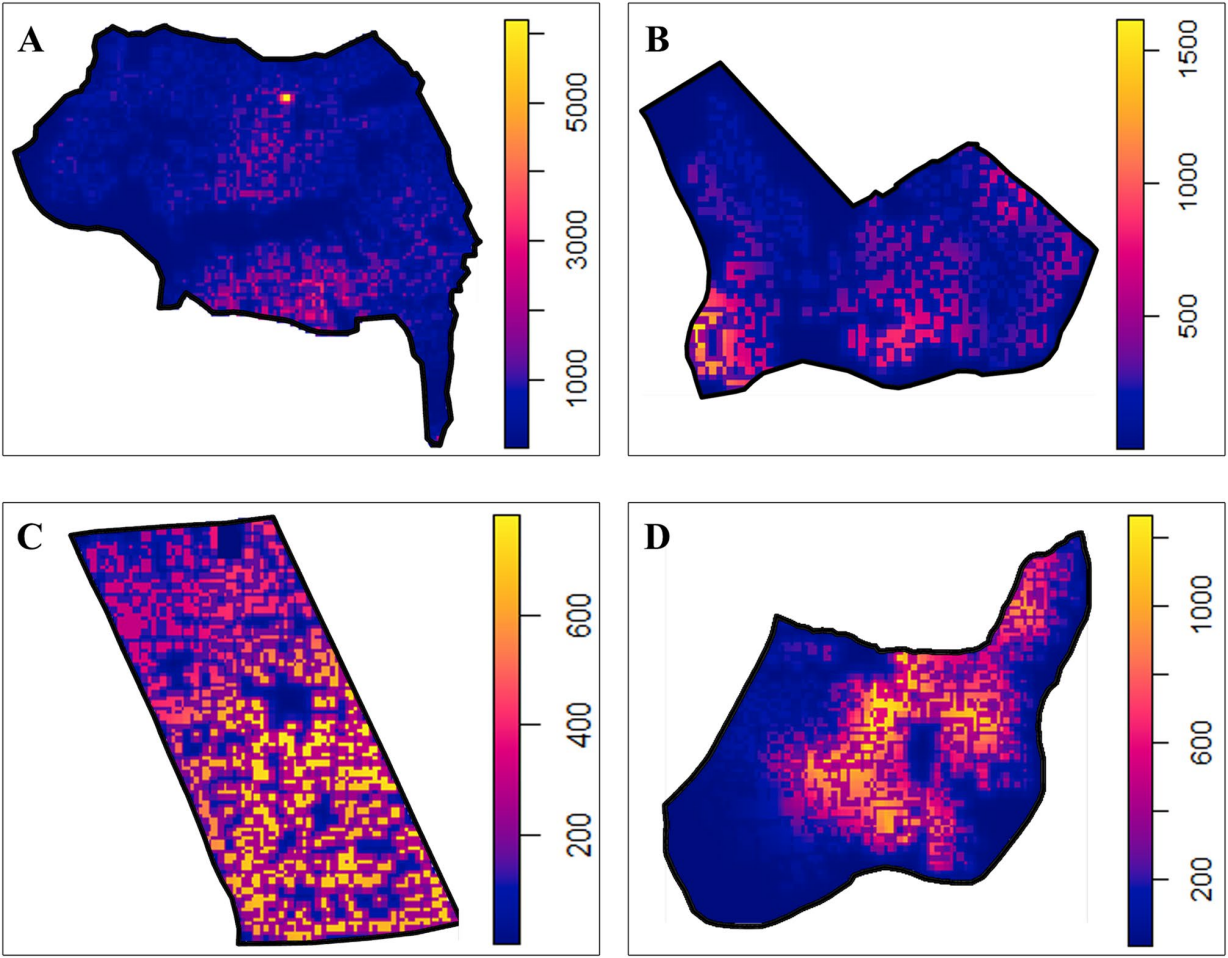
Predicted point pattern intensity for dengue incidences in the respective study areas. High risk localities were illustrated in the yellow colour while the low risk localities were illustrated in the blue. Variation of intensity levels are scaled adjacently to the intensity map of the study areas. (**A**) Eriyawetiya; (**B**) Akbar Town; (**C**) 3^rd^ Kurana; (**D**) Welikadamulla.

During the mathematical modelling of patient locations of dengue in the study areas using Poisson point pattern models, different point pattern intensities were predicted for dengue incidences in the study areas (Fig. 2). Further, coefficients obtained for the each variables in study areas were summarized in Table 2. The developed equations for each study area are available in Supplementary Table S1.

**Table 2:** Coefficients obtained by the Poisson point process modelling for the variables in different high risk study areas.

	Eriyawetiya	Akbar Town	3^rd^ Kurana	Welikadamulla
(Intercept)	8.811 (0.568)*	6.918 (1.424)*	5.029 (2.422)*	6.21 (1.162)*
Positive breeding locations	− 0.001 (0.001)	− 0.001 (0.001)	− 0.001 (0.001)	0 (0)
Roads	− 0.032 (0.007)*	− 6.677 (20.863)	5.483 (26.222)	28.572 (15.57)
Total buildings	− 0.076 (0.009)*	− 0.108 (0.025)*	− 0.093 (0.015)*	− 0.072 (0.015)*
Land use-home gardens	0.081 (0.022)*	− 0.015 (0.008)	− 0.922 (9.0683)	− 0.019 (0.005)*
Land use-marshy lands	− 0.004 (0.001)*	− 0.004 (0.004)	0.002 (0.001)	0.006 (0.002)*
Land use-urban areas	− 0.0001 (0)*	0.002 (0.002)	N/A	0.003 (0.001)*
Public places	− 0.004 (0.001)*	− 0.006 (0.003)*	− 0.002 (0.002)	− 0.002 (0.001)
Contour	0.043 (0.022)	0.001 (0.082)	0.185 (0.441)	− 0.224 (0.249)

*Significantly correlated layers with patient locations in respective Poisson point process models. Standard errors have indicated within parentheses. A 5% significance level was used to identify significant correlations. N/A-Urban areas are not included in the land use maps of 3^rd^ Kurana study area.

During the modelling, base intensity (Intercept) and total building were significant in all areas. Further, land uses were significant in Eriyawetiya and Welikadamulla study areas while public places were significant in Akbar Town as well as Eriyawetiya study areas.

**Figure 3:**
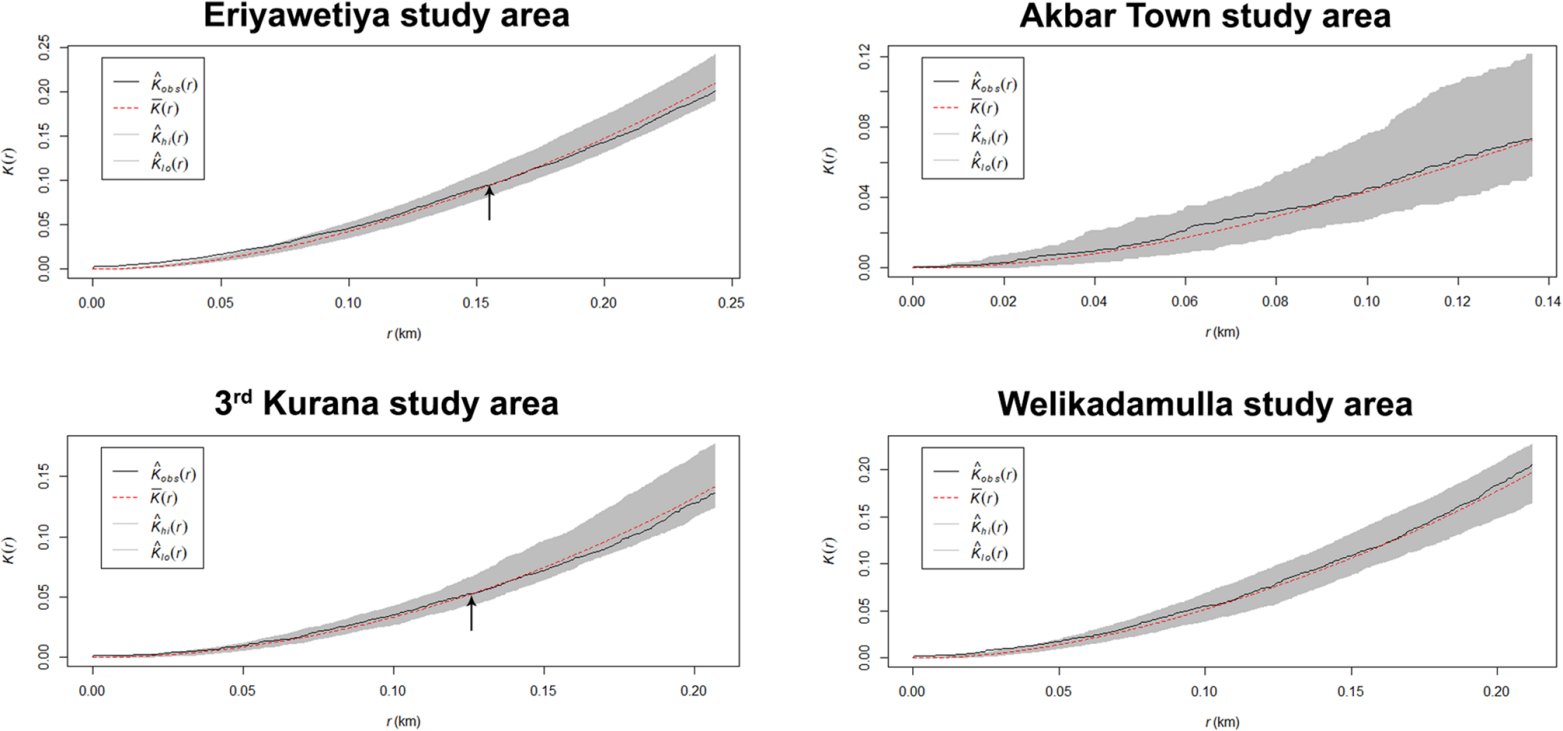
Observed Ripley’s K-functions and simulated envelopes for Poisson point process models in the study areas. $r$—The vector of values of the argument r at which the function K has been estimated; $\widehat{K}$—The estimates of $K(r)$; $obs$—Observed values of the summary function for the dengue patient location data pattern; $lo$—Lower envelope of simulations; $hi$—Higher envelope of simulations.

According to the observed Ripley’s K-functions (Fig. 3), in Eriyawetiya and 3^rd^ Kurana study areas, dengue incidences were clustered at some scales and be dispersed at others. Significant clustering is detected within a radius of approximately 155 m and 125 m in Eriyawetiya and 3^rd^ Kurana study areas, respectively and dispersion is observed afterwards.

### Identification of distribution of dengue incidences in the study high risk areas in different monsoon periods

**Figure 4:**
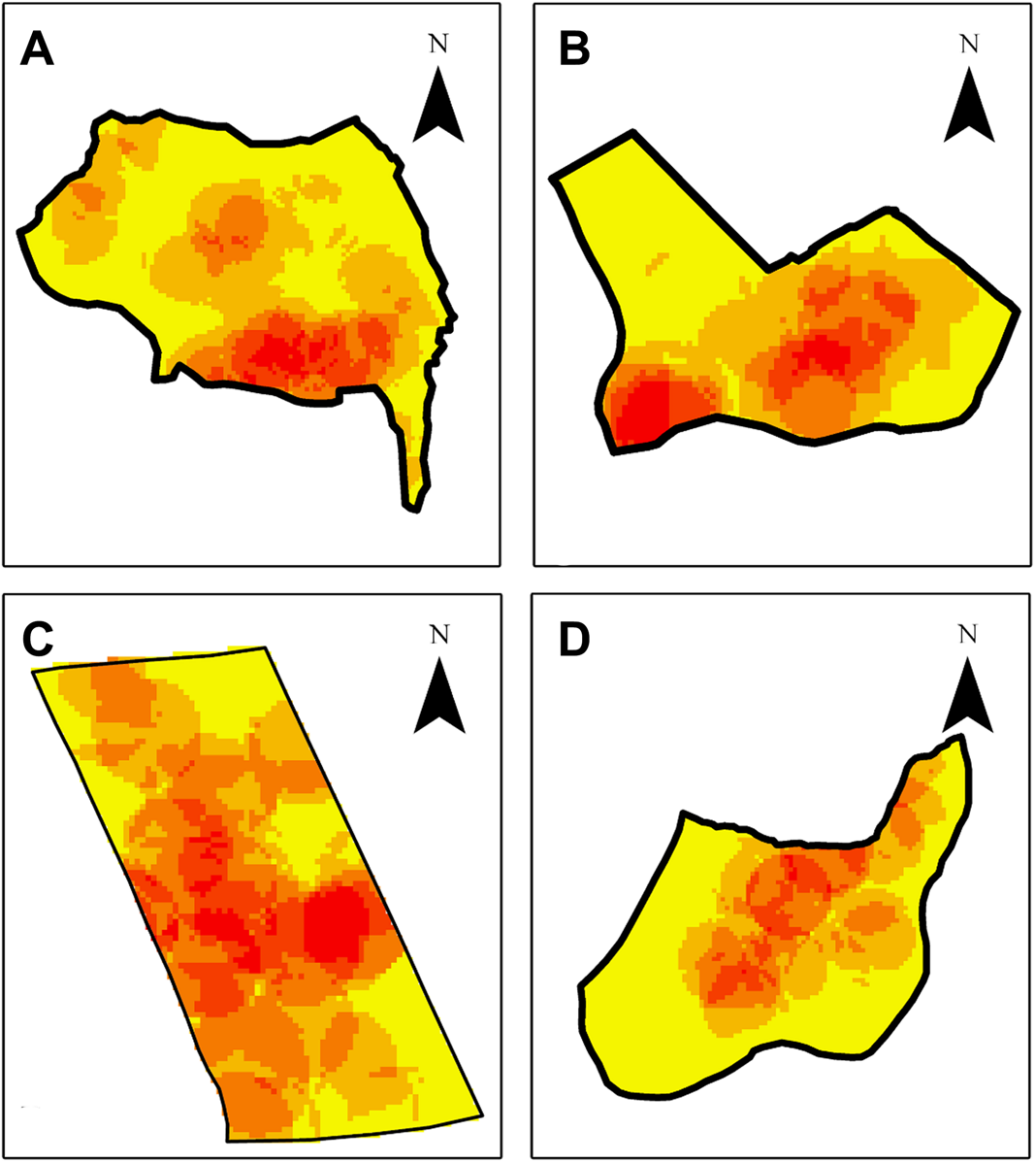
Distribution of dengue incidences in south-west monsoon period in the study areas. (**A**) Eriyawetiya; (**B**) Akbar Town; (**C**) 3^rd^ Kurana; (**D**) Welikadamulla. Distribution of dengue incidences in different monsoon periods in the study areas were illustrated in Supplementary Figs. S5–S8. Figure was generated using Esri ArcGIS 10.2.1.

In each study area, the dengue incidences were clustered depending on the monsoon periods and mapped separately to identify the distribution of dengue incidences in the study areas. In the study, the highest number of dengue incidences were reported during the south-west monsoon period. Figure 4 illustrated the heat maps of distribution of dengue incidences in the south-west monsoon period in the study areas. Distribution of dengue incidences in different monsoon periods in each study area were illustrated in the Supplementary Figs. S5–S8.

## Discussion

Present study aimed to develop a risk model to identify the risk localities in the dengue high risk areas. Kernel density and Euclidean distance based approaches are widely used in raster development of GIS modelling. Kernel density was used to fit a smoothly tapered surface to point layers while Euclidean distance was used to identify close exposures of polygon layers^17^. The risk values were ranked for each layer depending on their contribution to the transmission of dengue incidences. Based on the ILWIS Applications Guide^18^, the maximum risk value for the developed model was assigned as 10. Previous study conducted on mathematical modelling of dengue incidences in the Gampaha District have stated exponential influence of previous month cases on current month disease transmission in the district^19^. Further, investigation on adult and immature stages of dengue vector mosquitoes indicated that DENV are present in adult dengue vector mosquitoes and significant correlations of entomological indices with patient cases in the same district^20,21^. Therefore, patient locations and positive breeding container layers were selected as maximum risk variables and assigned the risk value of 10 as these variables are directly involved in disease transmission. In the modelling, dispersed risk distance patient cases and breeding places were selected less than average flight distance of dengue vector mosquitoes which is 400m^22^. Further, these study areas are considered to be highly congested areas and therefore, total building and home garden layers were given second highest ranking. Previous study conducted in Indonesia reported that consistent high number of dengue cases in larger areas of buildings even though the correlation is weak^23^. Further, higher dengue vector population densities were reported around home gardens from many countries^24–26^. Therefore, moderate risk level, risk value of 6, was assigned to total buildings and home garden layers. Recent study conducted in the Gampaha District demonstrated the contribution of daily commutes of people for transmission of dengue in the district^27^. When people visited to urban areas, there is higher probability of acquiring of dengue as these urban and suburban areas may act as dengue hot spots and artificial reservoirs which has been documented previously in Sri Lanka as well as other countries^28–31^. Therefore, land use layer for urban areas was given third highest ranking in the risk model. Another study conducted in Sri Lanka reported roads are important aspects for transmission of dengue^25^ and households in the present study areas located along main roads or have access roads. Further, previous study in Sri Lanka reported the potentials of public places play as artificial reservoirs dengue^32^ because of higher prevalence of breeding places around public places. It is a well-known fact that distribution of dengue vector mosquitoes varies with the elevation depending on geographical areas. Therefore, roads, public places and elevation layers were ranked in the third position with risk level of 4. However, the lower risk distances were assigned to road and contour layers as the layers are not related directly for transmission of the disease even though they play important role. Previous study conducted in Kenya and Uganda has reported higher dengue vector mosquito populations close to vegetation and marshy lands^33^ which may provide resting places of dengue vectors, especially for male mosquitos. When considering the study areas, with the exception of the 3^rd^ Kurana study area, all other study areas have close proximity to marshy areas and therefore these areas were included as a variable in the present study. Since it is not directly involving in mosquito population increase or disease transmission, the lowest rank was assigned to marshy areas of land use layers.

When comparing the generated risk maps with satellite imageries, vegetation covers were observed in high risk localities in all study areas. The reason could be the vegetation covers make better resting places for dengue vector mosquitoes. Even though the *Ae. aegypti* mosquitoes, the main vector of DENV, rest indoors^34^, previous studies conducted in Malaysia and Kenya reported the preference of *Ae. aegypti* to rest and breed outdoors due to increased breeding opportunities without affecting lifespan or gonotrophic activity^35,36^. Meanwhile, it is well-known fact that *Ae. albopictus*, the subsidiary vector of DENV, prefers vegetation to rest and breeding in both natural and man-made containers^37,38^.

When comparing the intensity maps generated from the Poisson point process model with generated risk maps, differences in localization of intensities were observed specially in Eriyawetiya and Welikadamulla study areas. In the risk map of Eriyawetiya study area, risk localities were located mainly along the roads in the area and this observation was even statistically significant in Pearson correlation analysis. However, when considering the intensity map from the Poisson point process model, lower predicted intensity was observed in most of the locations in the study area and high intensities were observed around the southern border along the Devasumithrarama road and in central area. When considering the Welikadamulla study area, even though risk map indicates that dengue is high virtually all over the area, the predicted intensity map illustrates that dengue may high in central and northern border of the area along the Welikadamulla road. Interestingly, while the dengue high intensity localities in both Eriyawetiya and Welikadamulla study areas are mainly used as home gardens, these localities have close proximity to crowded public places, such as schools, temples, community halls, etc. Perhaps, these public places may have acted as artificial reservoirs of dengue. This is further observation in the high density localities in Akbar Town and 3^rd^ Kurana study areas. In the Akbar Town area, high intensities were observed around mosques. In the 3^rd^ Kurana study area, many public places, such as schools and churches, are located in the central and southern area where intensities were high. However, the lowest dengue intensities were observed from the 3^rd^ Kurana study area.

In the Poisson point process model, highest intensity range was observed in the Eriyawetiya study area while the lowest was observed from 3^rd^ Kurana. Eriyawetiya study area is located close to the northern border of Colombo, the commercial capital in Sri Lanka, where highest number of dengue cases are reported in the country^39^. Recent study reported that human commutes to risk areas in Colombo and transportations may play significant role transmission of dengue in the nearby areas, such as Eriyawetiya study area, leading to higher intensities^21^. However, the overall lowest intensities reported from 3^rd^ Kurana study area may be due to continuous encouragement of dwellers in the area to remove dengue vector mosquito breeding places and use of protective measures by the churches and clergies.

The results of Pearson correlation analysis and Poisson multivariate point process model were also different especially with respect to positive breeding locations and roads layers. Positive correlation was observed between breeding places and patient locations in Pearson correlation analysis, which can be expected as dengue vector mosquitoes are anthropophilic mosquitoes with low flying ranges, were different from the results of Poisson point process model. In the model, no or negative correlation was observed between patient locations and breeding places. In a multivariate model, all explanatory variables are modelled to capture the true variation of the response variable while in Pearson correlation only one explanatory variable is considered at a time. The negative correlation in Poisson model with breeding places may be due to the hidden breeding places. These breeding places may be unidentified due to level of personal expertise, restrictions of accessibility to household, limitations due to inadequate resources, etc. which lead to differences between actual adult population and larval indices^21^. Further, even though road layers were shown similar behaviours for 3^rd^ Kurana and Welikadamulla study areas both in Pearson correlations and Poisson modelling, differences were observed in Eriyawetiya and Akbar Town. The positive correlations observed between patient locations and road layers could probably be because of high congestion of households alongside the roads and therefore, even single DENV infected mosquitos can spread the disease to all households as these mosquitoes probe many humans during blood feeding. Similar observation has been reported in previous study conducted in West Indies^40^. The study further states that more dengue cases being found within 1–3 km away from various types of roads. This may be the reason for the observed negative estimates from multivariate Poisson model in Eriyawetiya and Akbar Town study areas as the patient locations are very close to access roads.

When analysing the observed $K$ -functions of the developed Poisson multivariate models for the study areas, both clustering and dispersions were observed for Eriyawetiya and 3^rd^ Kurana study areas while only clustering was observed in the Akbar Town and Welikadamulla areas. Interestingly, in Eriyawetiya and 3^rd^ Kurana study areas, clustering was observed a radius of approximately 150 m. This is comparable to the general flying range of dengue vector mosquitoes, especially with regards to the *Ae. aegypti*^41^, the main dengue vector mosquito. Further, this may be an indicative of that patients in a small areal cluster are prompted due to a single infected dengue vector mosquito. During the analysis, both isotropic^42^ and translation^43^ edge correction methods were considered, therefore, edge effects arising from the unobserved patient locations outside study area can be hampered when estimating the $K$-functions. The estimations of $K$-functions were within the upper and lower envelopes of simulated functions in Akbar Town, 3^rd^ Kurana and Welikadamulla study areas, that is, given particular distance, the data and simulated patterns were statistically equivalent. This indicates that dengue patient locations in the study areas were undergone a complete random pattern or CSR except for Eriyawetiya study area. This observation is further confirmed by the results of Maximum Absolute Deviation (MAD) and the Diggle-Cressie-Loosmore-Ford (DCLF) non-graphical tests^44^.

Among four monsoon seasons, the first inter-monsoon season occurs during March and April months. The Southwest monsoon period starts in May and it lasts till September. During the October and November, the second inter-monsoon period occurs and the Northeast monsoon lasts for three months from December to February. When analysing the distribution of dengue incidences in the monsoon periods, the highest number of dengue incidences were reported from the Southwest monsoon period in all study areas. The Gampaha District is located in the western part of Sri Lanka and during the monsoon period, the district experiences a rainfall of 750–2000 mm. In other monsoon periods, rainfall of the Gampaha District is less than 1000 mm^45^. The reason for higher precipitation in the Southwest monsoon period includes the presence of abundant water bodies, such as Arabian Sea and Indian Ocean, leading to higher accumulation of moisture in Southwest monsoon winds^46^. The higher rainfalls increase not only the availability of the breeding containers for dengue vector mosquitoes, but also favourable environmental conditions, viz. humidity and temperature, for its development. This will lead to increased disease transmission during the Southwest monsoon season compared to other monsoon seasons.

The developed models can be used to identify risk localities easily for healthcare workers and decision makers. The Poisson point process models can be developed using freely available software and packages. Further, road maps can be easily obtained for freely available sources and modified easily using freely available GIS software. With the advantages of technology, correct GPS locations of positive dengue vector mosquito breeding places and patients can be easily obtained using mobile devices with minimum wage during vector control programmes and export directly into GIS software. Since roads, land use, buildings and contour being not changing frequently in a particular area, with the aid of available data on patient locations as well as positive breeding places, it is possible to develop risk maps monthly or biannually to assess the risk levels of high risk areas. Further, when health authorities have risk map of particular area over few years, then it is possible to identify risk localities and transmission of dengue in an area in advance. This is particularly important in outbreaks and epidemic progression, so that they can have a better scenario of undergone situation to use scarce health resources effectively to control disease transmission. Meantime, the model can be further enhanced by incorporating serotype data which may lead identify index cases and initial clusters. A combined approach of predictive mathematical models^19^ and genetic approaches to identify the virulence of circulating dengue viruses^21^ will provide sufficient information for health authorities to take timely actions, such as intensive source reduction programmes, targeted intervention programmes or deploy vector reduction tools such as ovitraps^9^, to manage the situation to prevent propagation of outbreaks and epidemics.

## Conclusions

The developed GIS-based model can be utilized easily to identify risk localities at early stage in high risk areas which is not available yet in the Gampaha District as well as in Sri Lanka. In the high risk areas of current study, clustering of dengue incidences were observed at a radius of 150 m. The output of the developed model can be used as an early warning tool to explore and identify the current situation of dengue in an area providing valuable insights for healthcare authorities to understand disease propagation patterns and allocate scarce public health resources effectively to prevent impending dengue outbreaks and epidemics. When the GIS-based model coupled with mathematical modelling and phologenetic approaches, it will probably illustrate better scenario of present situation dengue outbreaks in real-time in risk areas. When these model apply over a large area or at national level in time-series manner, transmission patterns of dengue may be identified easily.

## Methods

### Study area

The Gampaha District, expands over 1387 km^2^, is located near the Northern border of the District of Colombo. Gampaha District is the second most populated district in Sri Lanka. Fifteen Medical Officer of Health (MOH) areas, sectioned into 106 Public Health Inspector (PHI) areas and 1177 Grama Niladhari (GN) divisions with 1784 villages are located in the district^47^. Previous study conducted on the mathematical modelling of dengue incidences with meteorological variables reported that the Kelaniya, Wattala and Negombo MOH areas were being with highest dengue prevalence in the district^19^ and these MOH areas together with Mahara MOH area, where a moderate risk area which has close proximity to Kelaniya and Wattala MOH areas were selected for the current study. The highest number of dengue incidences reported GN division of each selected MOH area was selected as the study areas of the present study (Supplementary Fig. S9).

### Data collection

The baseline map for Sri Lanka was obtained from the Survey Department of Sri Lanka as a shapefile. GIS-based models were prepared to develop risk maps with the aim of identification of risk areas in study sites. During the development of risk models, locations of patients and positive breeding containers were collected using consumer grade GPS receivers for all study areas from January, 2014 to June, 2018. The shapefile for GN division boundary maps for the Gampaha District were downloaded from the Survey Department of Sri Lanka and land use maps for the study areas were collected from the Land Use Policy Planning Department, Sri Lanka as separate shapefiles. The 1:10,000 digital elevation maps for the Gampaha District was obtained from the Survey Department.

### Development of GIS-based model

Geographical information system-based models and risk maps were developed using entomological, environmental/ecological and social factors affecting transmission of dengue for the Gampaha District using ArcGIS 10.2 software (ESRI Inc., USA) and layer creations and transformations were performed using the QGIS 2.18-Long-term Release (LTR), QGIS Development Team).

**Figure 5:**
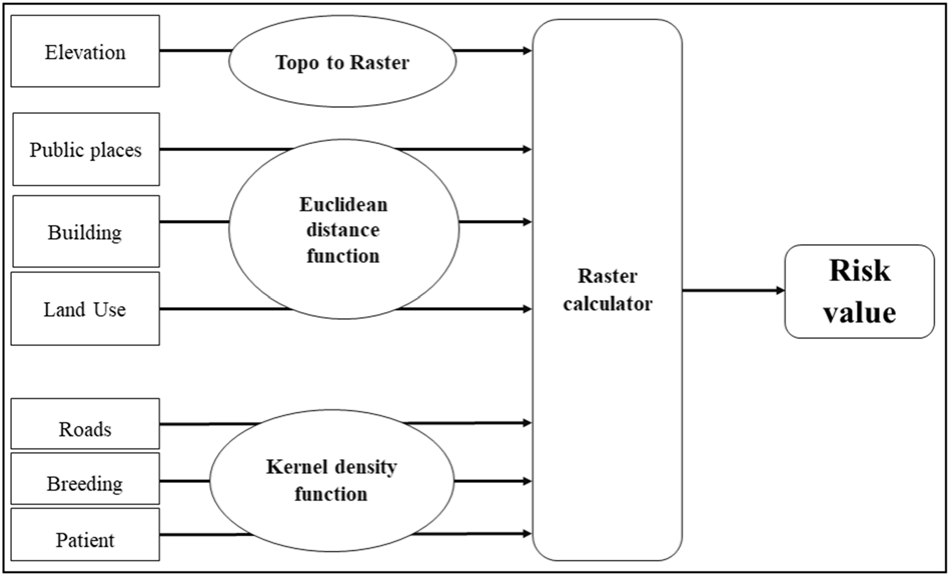
Systematic flow chart of risk model development.

GIS-based risk models were prepared to identify the risk areas and factors in the study areas in the Gampaha District. Separate layers were developed representing locations of dengue patients and positive breeding containers of *Aedes* dengue vector mosquitoes, roads, land use, total buildings, public places and elevations in each study area. The schematic structure of the underlined model is illustrated in Fig. 5.

#### Georeferencing and projection transformations

An image of MOH areas in the Gampaha District was georeferenced to develop the MOH area map of the district and saved as separate shapefile and transformed into Universal Transverse Mercator (UTM) zone 44 N (EPSG:32644) coordinate system^48^. The GPS readings on patient locations and positive breeding places were converted to UTM coordinate system (EPSG:32644) prior to saving as separate layers for each study area. Building and public places layers for each study area were created based on satellite imageries of USGS EarthExplorer^49^ and verified using Google Maps. Road maps for the study areas were developed based on the maps available at OpenStreetMap^50^ and verified using Google Earth. Separate shapefiles were created for urban areas, homesteads and marshy lands using the land use files for each study area. The digital elevation maps was clipped to extract the elevation levels of the study areas. All generated shapefiles for each study area were transformed into UTM zone 44 N coordinate system prior to model building.

#### Development of GIS-based model to generate risk maps to identify dengue risk in the study areas

The development process of the GIS-based model for study areas were summarized in the Supplementary Fig. S10. Raster layers were developed separately for patient data, breeding places and road maps layers using the kernel density function. The Euclidean Distance function was used to generate raster layers for different land uses, buildings and public places. Raster layers were developed for contour layer using the ‘Topo to Raster’ function. Based on previous literature^51^, risk values were assigned prior to assigned (Table 3).

**Table 3:** Risk values assigned for study layers during the development of the model in each dengue study area.

Study layer	Risk value	Distance (m)
Patient incidence locations	10	300
Positive breeding containers	10	100
Contour	4	20
Roads	4	50
Total buildings	6	450
Public places	4	500
Land use-Urban areas	5	1500
Land use-Home gardens	6	450
Land use-Marshy lands	2	200

#### Identification of correlations between patient cases with developed layers in the study areas

Spatial correlations of each layer to patient cases were studied by introducing specified number of random point features to the study area layer. Minimum Allowed Distance parameter was then calculated to each raster using the random point layer. The distances calculated for ‘patient density’ raster was considered as the dependent variable and distances calculated for each layer were considered as explanatory variables. Pearson correlation was performed to identify significantly correlated layers.

#### Mathematical modelling of patient cases with developed layers in the study areas

Spatial Poisson point process model was fitted considering patient locations as a function of developed raster layers developed from breeding places, road maps, different land uses, buildings and public places layers. The analysis was performed in R statistical software^52^ using *raster*^53^ and *spatstat*^54^ packages for study areas. All the spatial objects were rescaled into kilometres prior to modelling. The equation for the model was:

1 $$\begin{aligned} \ln \lambda \left( i \right) &= \alpha + \beta_{1} \left( {Breeding\;Places} \right) + \beta_{2} \left( {Roads} \right) + \beta_{3} \left( {Total\;Buildings} \right) \\ & \quad + \beta_{4} \left( {Land\;Use_{Home\;Gardening} } \right) + \beta_{5} \left( {Land\;Use_{Marshi\;Lands} } \right) \\ & \quad + \beta_{6} \left( {Land\;Use_{Urban\;Areas} } \right) + \beta_{7} \left( {Public\;Places} \right) + \beta_{8} \left( {Contour} \right) \\ \end{aligned}$$

where $\lambda (i)$ is the modelled point pattern intensity for dengue incidences at location $i$ in the study area, $\alpha$ is the base intensity derived from the multivariate model and ${\beta }_{1}$ to ${\beta }_{8}$ are the estimated coefficients for each respective variable.

Upon developing, model outputs were plotted as predicted intensity of dengue in the areas to identify high-risk localities. Further, clustering pattern was also estimated using $K$-functions (Eq. 2)^55^, for the developed models in each study area, of 1000 pointwise Monte Carlo simulated realisations of Complete Spatial Randomness (CSR) with both isotropic and translation edge corrections.

2 $$\hat{K}\left( r \right) = \frac{a}{{n\left( {n - 1} \right)}} \sum\limits_{i} {\mathop \sum \limits_{j} I \left( {d_{ij} \le r} \right)e_{ij} }$$

where $a$ is the size of the study area, $n$ is the number of data points, and the sum is taken over all ordered pairs of points $i$ and $j$ in X. Here $d[i,j]$ is the distance between the two points, and $I(d\left[i,j\right]\le r)$ is the indicator that equals $I$ if the distance is less than or equal to $r$. The term $e[ij]$ is the edge correction weight.

### Development of heat maps to identify the distribution of dengue incidences with climate seasons

Sri Lanka experiences four climate seasons annually. The distribution of dengue incidences in the study areas were mapped separately in order to identify the distribution patterns of dengue incidences for each climatic season in the study areas. Heat maps were developed using the density tool for point data in ArcGIS software with output cell size of 10 m^56^. The number of dengue incidences reported during different climatic seasons was used to identify seasonal variations of dengue incidences in the study areas during the study period.

## Supplementary Information

Supplementary Information 1.
