# Supplementary material for: Multivariate spatio-temporal approach to identify vulnerable localities in dengue risk areas using Geographic Information System (GIS)
Source: Sci Rep. 2021 Feb 18;11:4080. doi: 10.1038/s41598-021-83204-1 (PMC7892844; doi:10.1038/s41598-021-83204-1)
Supplement: Supplementary file 1 — Supplementary Information 1. [file 41598_2021_83204_MOESM1_ESM.pdf]

Multivariate spatio-temporal approach to identify vulnerable localities in dengue risk areas using Geographic Information System (GIS)

Gayan P. Withanage[1], Malika Gunawardana[2], Sameera D. Viswakula[3], Krishantha Samaraweera[4], Nilmini S. Gunawardena[1], Menaka D. Hapugoda[1]*

[1]Molecular Medicine Unit, Faculty of Medicine, University of Kelaniya, Ragama, Sri Lanka
[2]Postgraduate Institute of Science, University of Peradeniya, Peradeniya, Sri Lanka
[3]Department of Statistics, Faculty of Science, University of Colombo, Colombo 07, Sri Lanka
[4]Epidemiology Unit, Office of the Regional Director of Health Services, Gampaha, Sri Lanka

Abstract

This is the supporting information for "Multivariate spatio-temporal approach to identify vulnerable localities in dengue risk areas using Geographic Information System (GIS)". It includes the study areas, the arrangement of the model developed using ArcGIS, comparison of ArcGIS-based model outputs with satellite imageries of study sites, significant variables and equations developed in Spatial Poisson point pattern model of each study are and variations of dengue incidences in different climatic seasons.

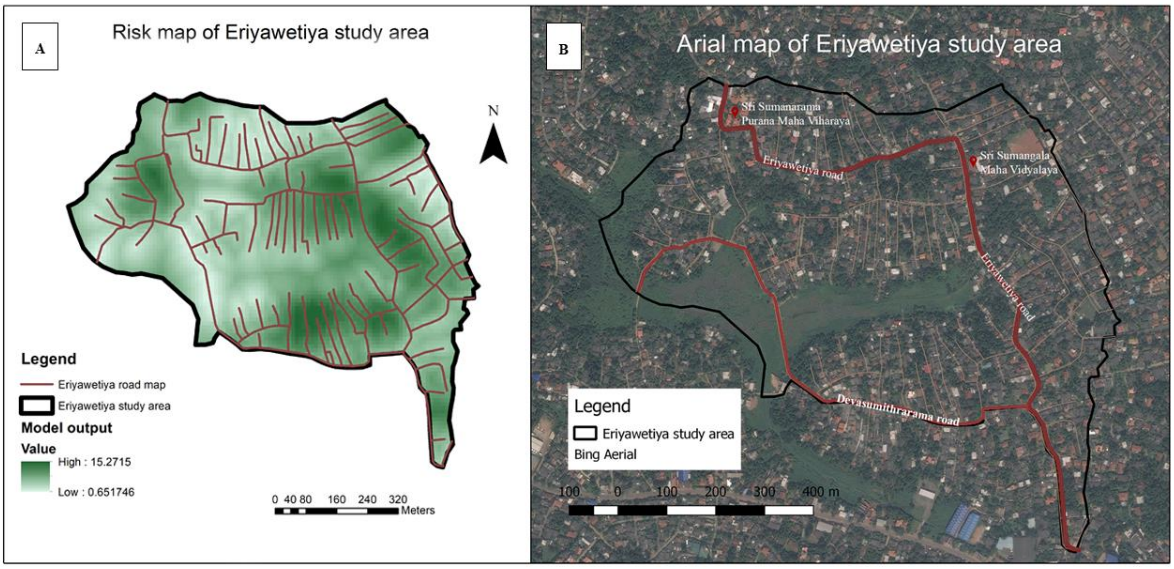

Figure S1. Generated risk map for the Eriyawetiya study area. A-Risk map. B-Satellite imagery. High risk localities were illustrated in the dark green colour while the low risk localities were illustrated in white. Risk map was composed using Esri ArcGIS 10.2.1.3497. Arial map was prepared using US Global Survey EarthExplorer satellite images which are freely available at https://earthexplorer.usgs.gov/.

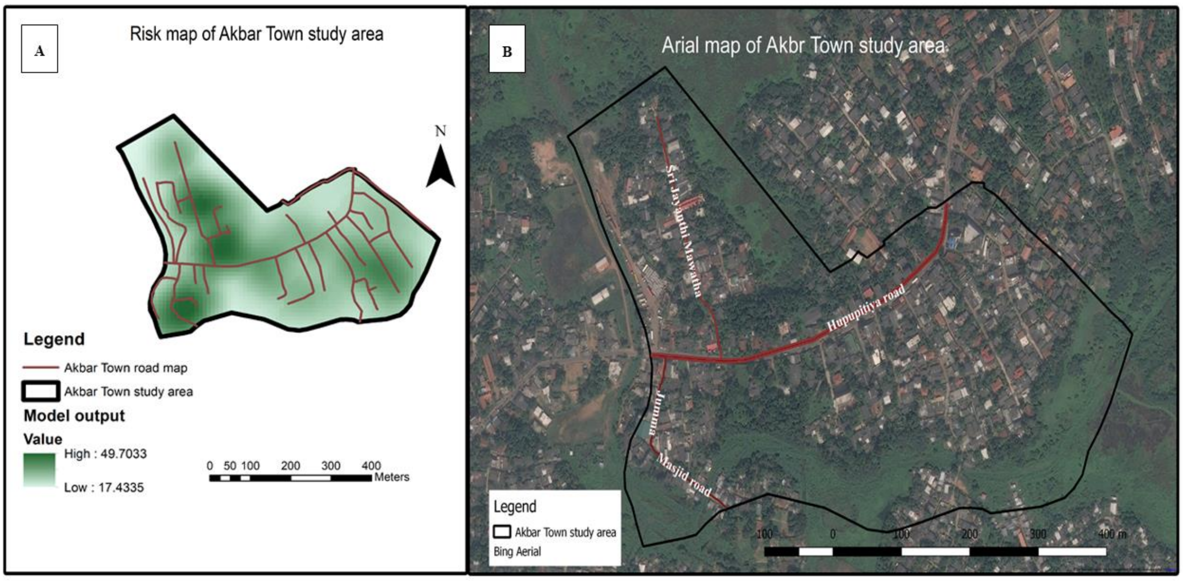

Figure S2. Generated risk map for the Akbar Town study area. A-Risk map. B-Satellite imagery. High risk localities were illustrated in the dark green colour while the low risk

localities were illustrated in white. Risk map was composed using Esri ArcGIS 10.2.1.3497. Arial map was prepared using US Global Survey EarthExplorer satellite images which are freely available at https://earthexplorer.usgs.gov/.

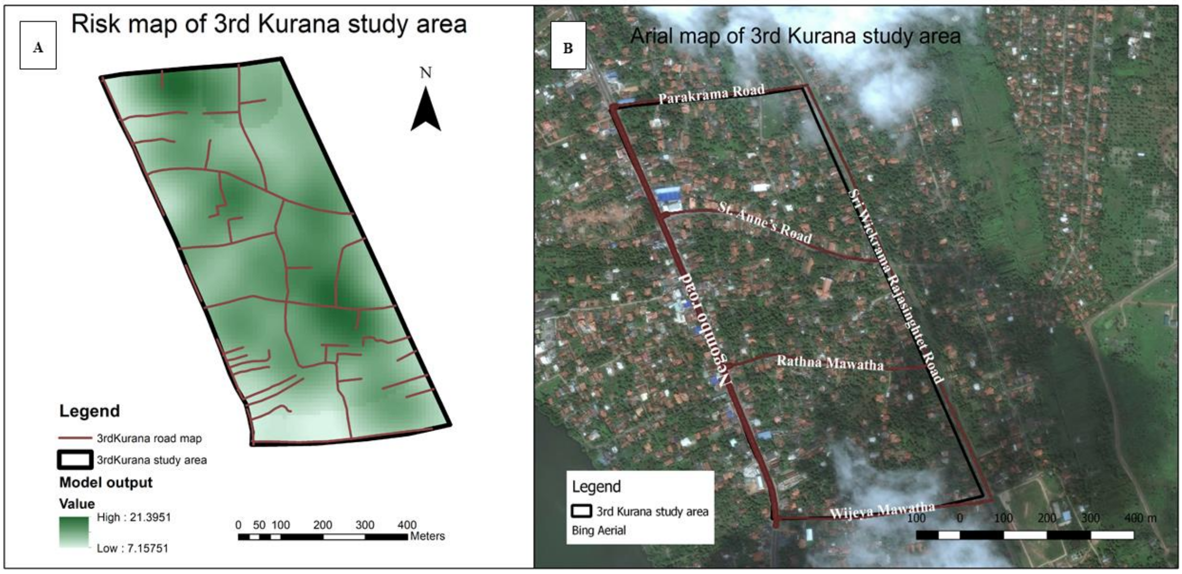

Figure S3. Generated risk map for the 3rd Kurana study area. A-Risk map; B-Satellite imagery. High risk localities were illustrated in the dark green colour while the low risk localities were illustrated in white. Risk map was composed using Esri ArcGIS 10.2.1.3497. Arial map was prepared using US Global Survey EarthExplorer satellite images which are freely available at https://earthexplorer.usgs.gov/.

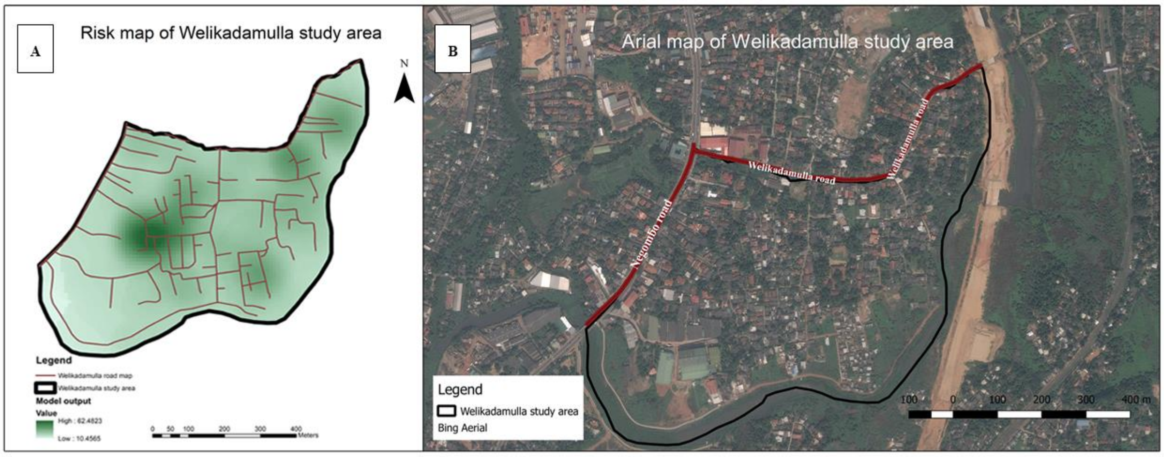

Figure S4. Generated risk map for the Welikadamulla study area. A-Risk map; B-Satellite imagery. High risk localities were illustrated in the dark green colour while the low risk localities were illustrated in white. Risk map was composed using Esri ArcGIS 10.2.1.3497. Arial map was prepared using US Global Survey EarthExplorer satellite images which are freely available at https://earthexplorer.usgs.gov/.

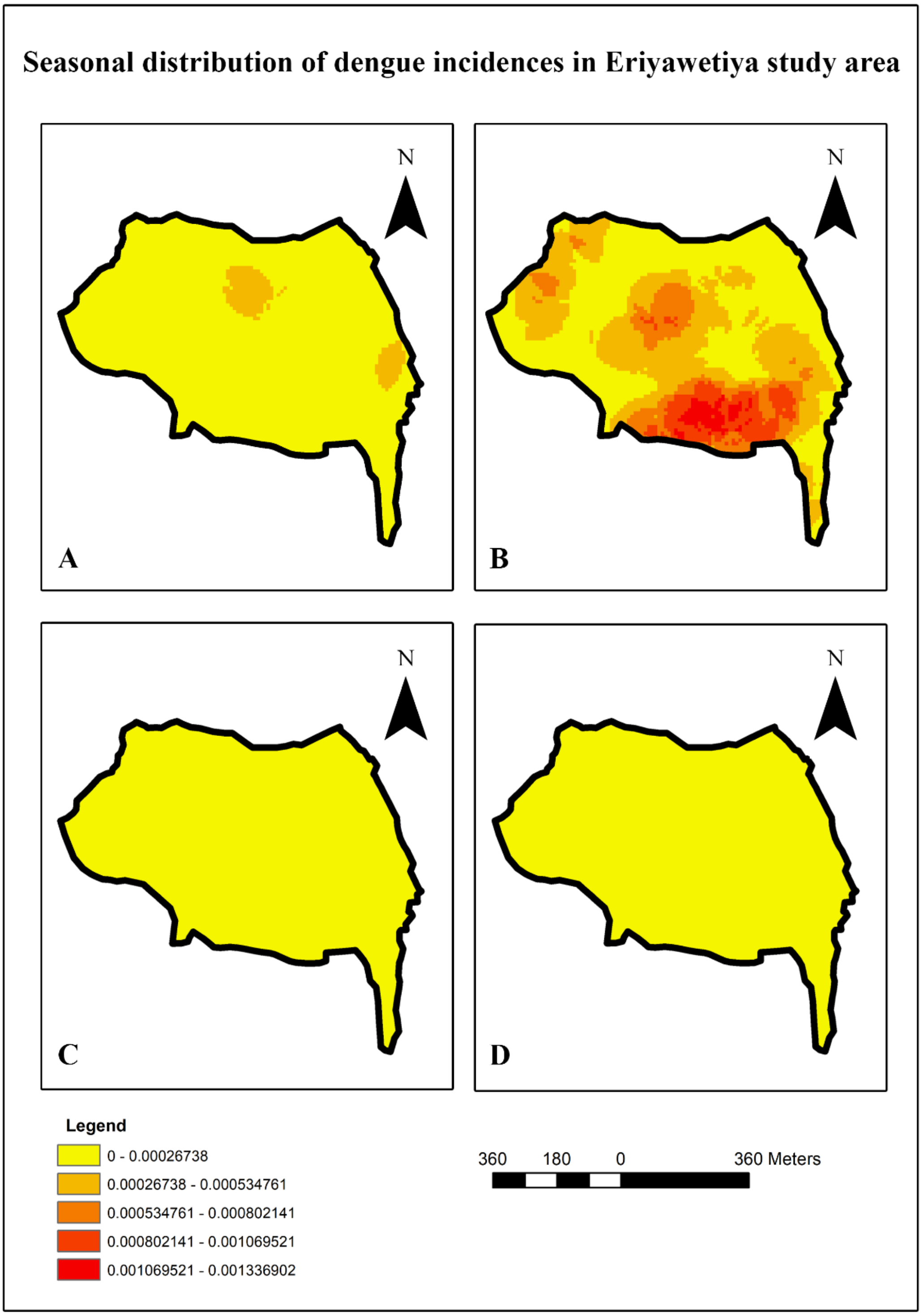

Figure S5. Distribution of dengue incidences in different monsoon periods in the Eriyawetiya study area. A - First inter monsoon season; B - Southwest monsoon season; C- Second inter monsoon season; D - Northeast monsoon season.

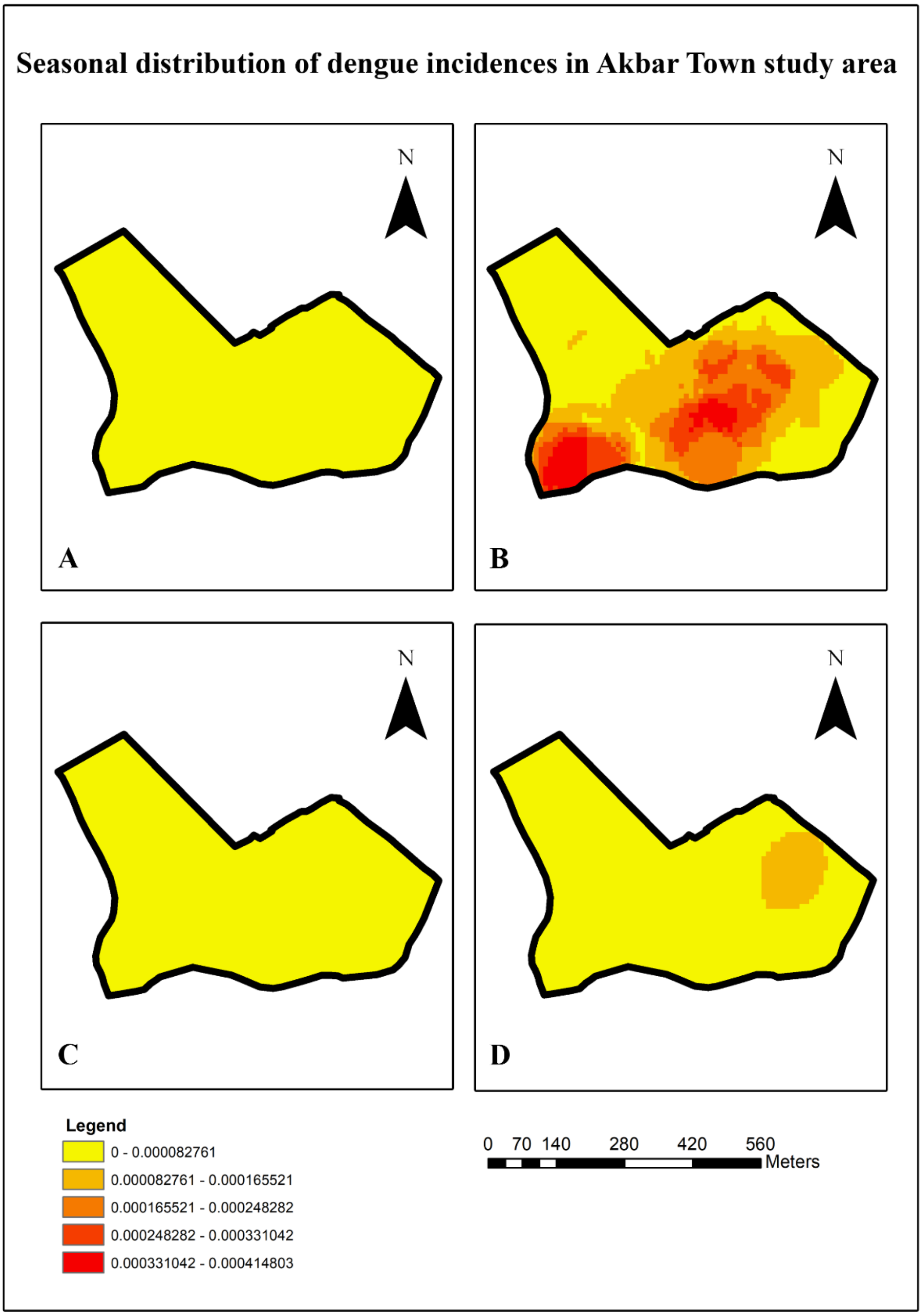

Figure S6. Distribution of dengue incidences in different monsoon periods in the Akbar Town study area. A - First inter monsoon season; B - Southwest monsoon season; C- Second inter monsoon season; D - Northeast monsoon season.

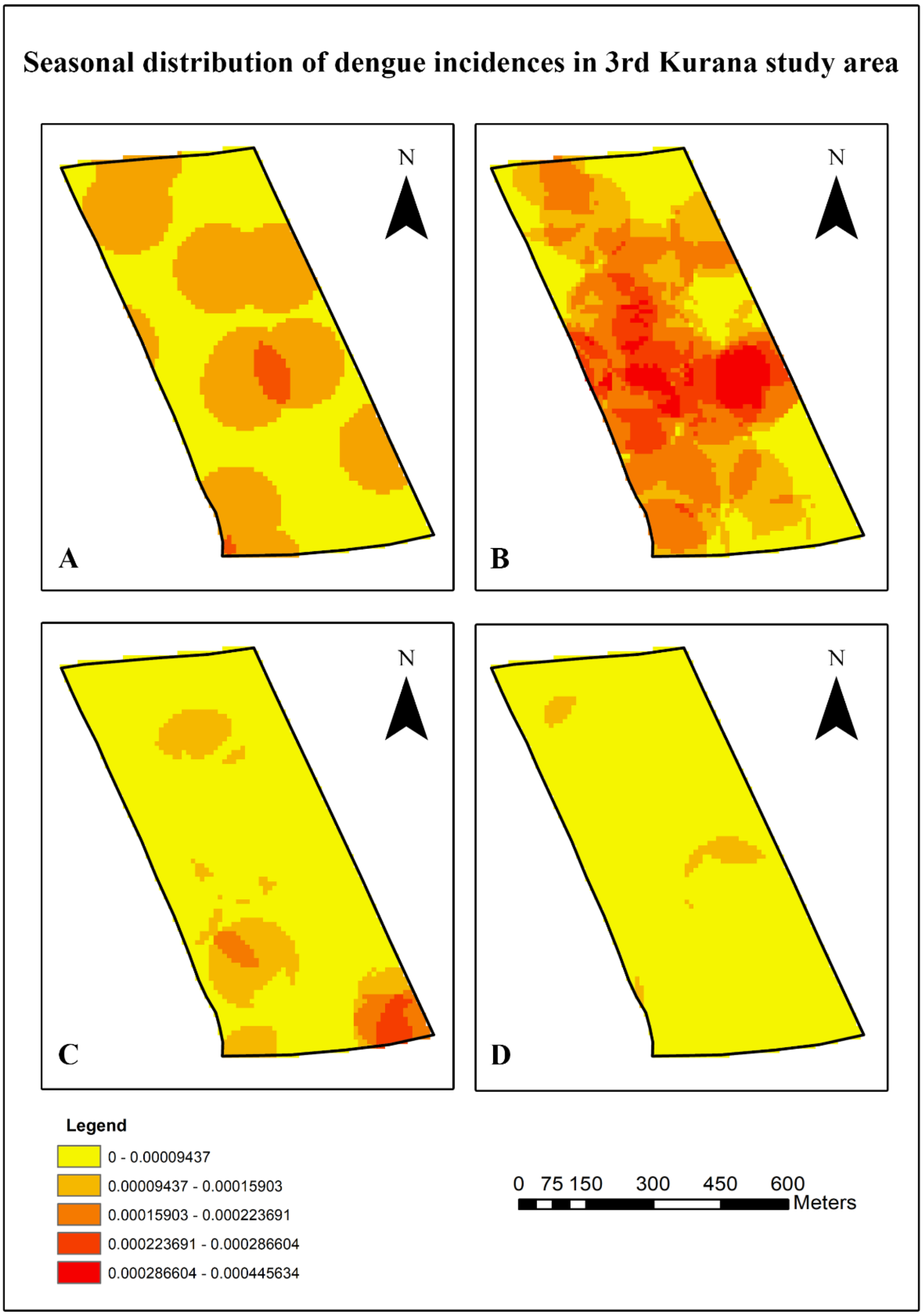

Figure S7. Distribution of dengue incidences in different monsoon periods in the 3rd Kurana study area. A - First inter monsoon season; B - Southwest monsoon season; C- Second inter monsoon season; D - Northeast monsoon season.

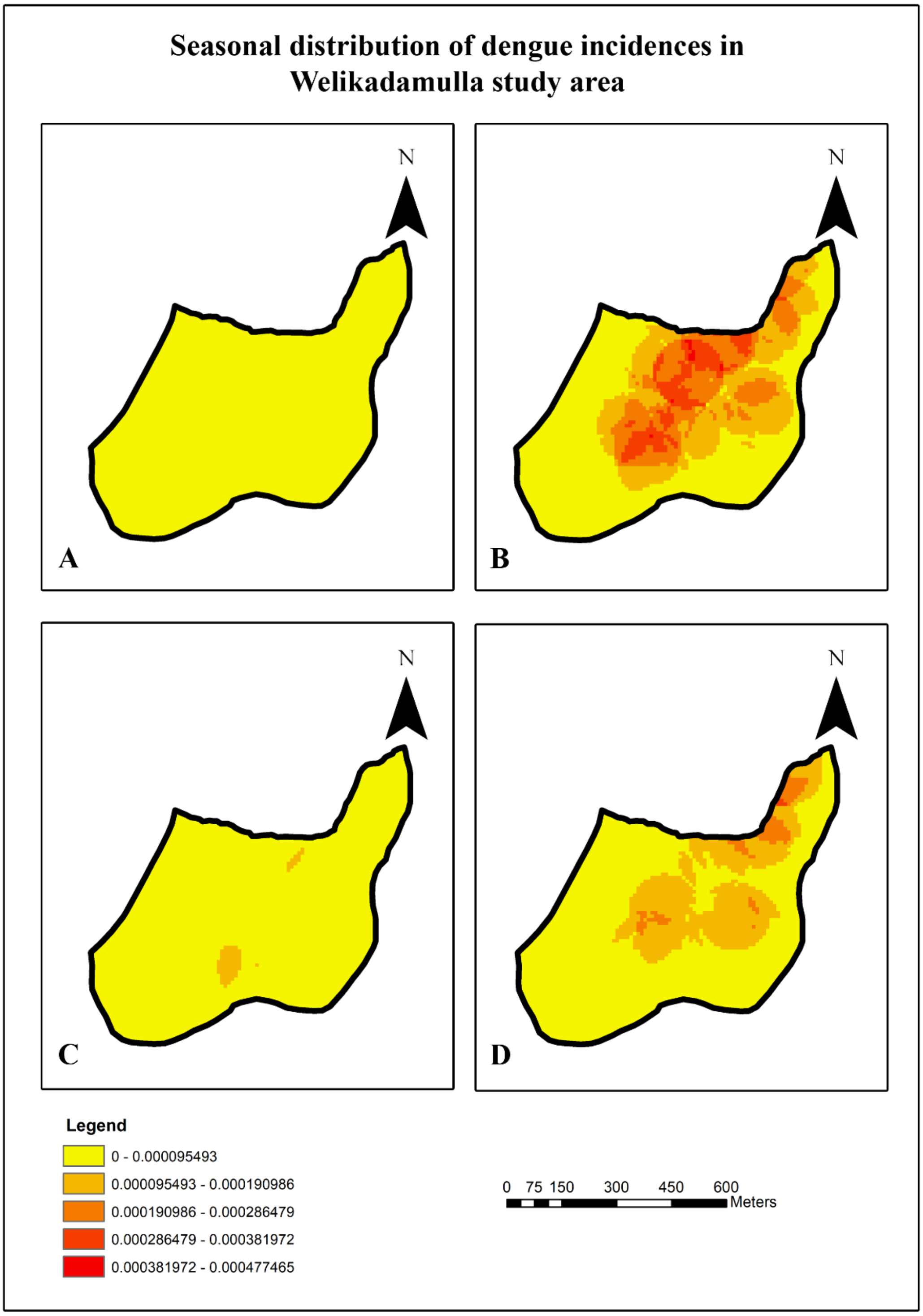

Figure S8. Distribution of dengue incidences in different monsoon periods in the Welikadamulla study area. A - First inter monsoon season; B - Southwest monsoon season; C- Second inter monsoon season; D - Northeast monsoon season.

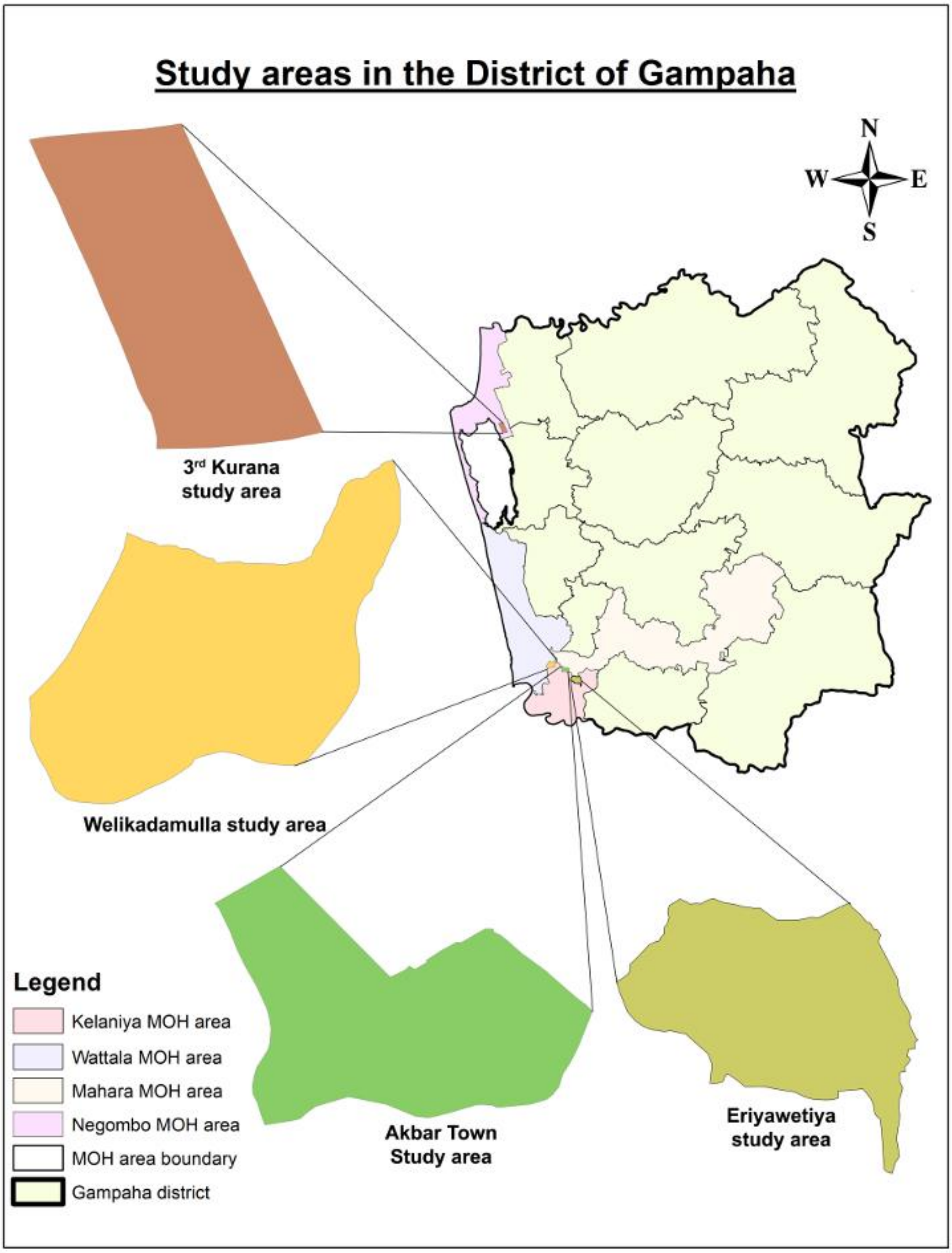

Figure S9. Study areas in the Gampaha district. The district and study areas maps were developed using the shapefile freely available for the Gampaha District at the website of Survey Department, Sri Lanka (http://www.survey.gov.lk/gn_updating/) and the figure was generated using Esri ArcGIS 10.2.1.3497.

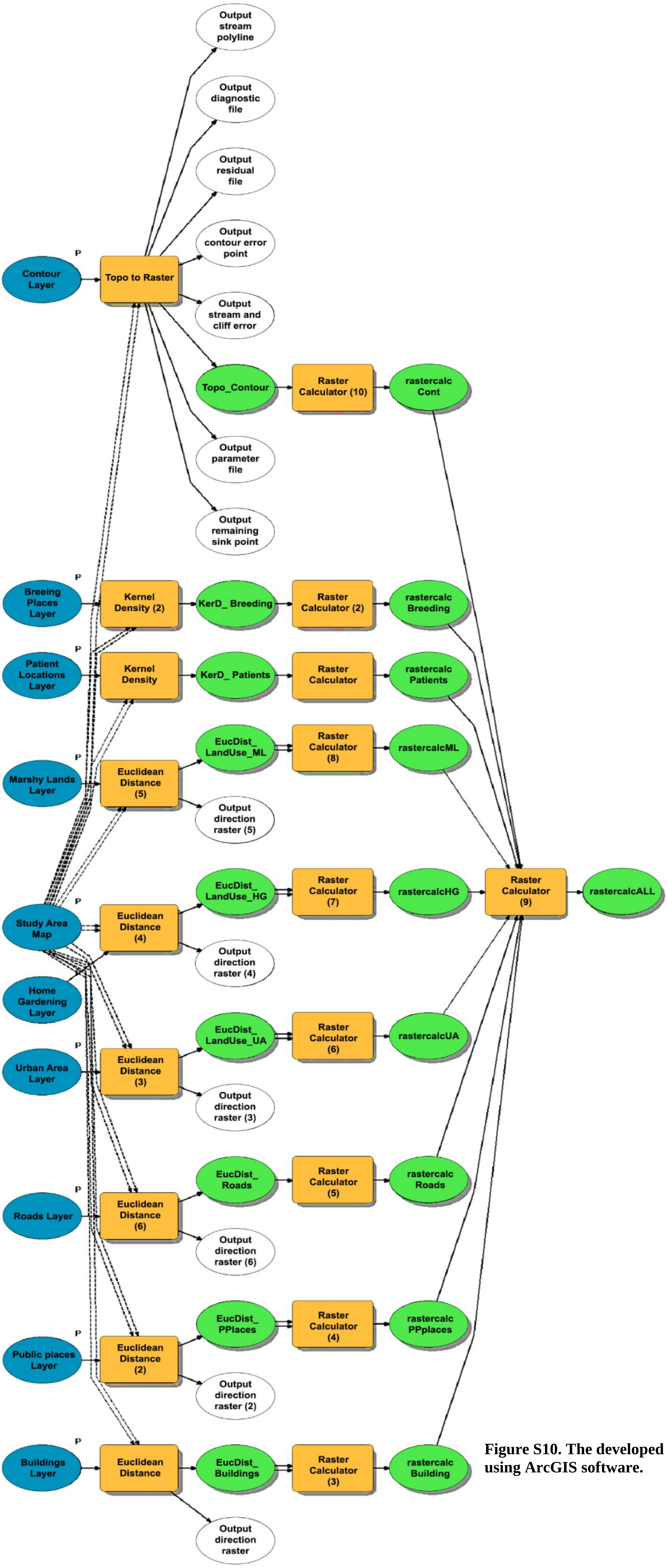

Figure S10. The developed model using ArcGIS software.

Study area	Spatial Poisson point pattern model equation
Eriyawetiya	$\ln\lambda(i) = 9.347 - 0.033(Roads) - 0.077(TotalBuildings) + 0.076\left(LandUse_{HomeGardening}\right) - 0.004(LandUse_{MarshiLands}) - 0.001(LandUse_{UrbanAreas}) - 0.004(PublicPlaces)$
Akbar Town	$\ln\lambda(i) = 6.402 - 0.103(TotalBuildings) - 0.004(PublicPlaces)$
3rd Kurana	$\ln\lambda(i) = 6.277 - 0.089(TotalBuildings)$
Welikadamulla	$\ln\lambda(i) = 5.958 - 0.072(TotalBuildings) - 0.024\left(LandUse_{HomeGardening}\right) + 0.006(LandUse_{MarshiLands}) + 0.001(LandUse_{UrbanAreas})$

Table S1. Spatial Poisson point pattern model equations for study areas with significant variables
